# Supplementary material for: Content-rich biological network constructed by mining PubMed abstracts
Source: BMC Bioinformatics. 2004 Oct 8;5:147. doi: 10.1186/1471-2105-5-147 (PMC528731; doi:10.1186/1471-2105-5-147)
Supplement: Additional File 5 — The original Chilibot query results of the term "long-term potentiation (LTP)" and 22 other terms, limiting the latest references analyzed to the years 1990, 1995, 2000, and 2004. [file 1471-2105-5-147-S5.bz2 › chilibotAdditionalFile5/ltp2000/html/CREB_CAMKII.html]

 


 **CREB** and **CAMKII** 
  
Found 10 abstracts in PubMed,  **10 abstracts were retrieved and analyzed**.  


---

 Search Google  |
 PDF files only 
|  EDU domain only 

---

**Interactive relationship** (e.g. stimulation, inhibition, etc)

- When Ser142 was mutated to alanine,  **CREB**  was activated by  **CaMKII** , as well as by CaMKIV.  Ref: 7958915 Genes Dev, 1994
- Thus,  **CamKII**  confers a dominant inhibitory effect on transcription by preventing dimerization of  **CREB** , and this mechanism may account for the attenuation of gene expression.  Ref: 11013247 J Biol Chem, 2001
- **CREB**  in the PSD fraction was phosphorylated both by protein kinase A and calcium calmodulin dependent protein kinase II  **CaMKII**  endogenous to the fraction, and dissociated from the PSD after phosphorylation, especially under  **CaMKII**  activated conditions.  Ref: 9795144 Brain Res Mol Brain Res, 1998
- **CamKII**  modified  **CREB**  binds to DNA efficiently as a monomer.  Ref: 11013247 J Biol Chem, 2001
- ...  **CaMKII**  phosphorylates  **CREB**  at Ser133 and a second site, Ser142.  Ref: 7958915 Genes Dev, 1994
- Here we show that phosphorylation of serine 142 in  **CREB**  by  **CamKII**  leads to dissociation of the  **CREB**  dimer without impeding DNA binding capacity.  Ref: 11013247 J Biol Chem, 2001
- Because both  **CaMKII**  and CaMKIV can phosphorylate  **CREB** , we pursued further the mechanism by which  **CaMKII**  and CaMKIV differentially regulate  **CREB**  activity.  Ref: 7958915 Genes Dev, 1994
- Transient transfection studies revealed that phosphorylation of Ser142 by  **CaMKII**  blocks the activation of  **CREB**  that would otherwise occur when Ser133 is phosphorylated.  Ref: 7958915 Genes Dev, 1994

**Parallel relationship** (e.g. studied together, co-existance, homology, etc.)

- These observations show that nociceptive stimuli activate a cholecystokinin pathway from the parabrachial nucleus to the ventromedial hypothalamus that may be important for homeostatic responses to tissue damage, and point to a putative intracellular route for calcium mediated FOS transcription via  **CaMKII**  and  **CREB**  for the regulation of ppCCK transcription.  Ref: 9766403 J Comp Neurol, 1998
- The molecular mechanisms that underlie plastic changes in the barrel cortex still remain unclear, although alpha  **CamKII**  and to lesser extend alpha beta  **CREB**  appear to be involved.  Ref: 9949558 Acta Neurobiol Exp (Wars), 1998
- The detection of spatial novelty is associated with a sequential activation of PKA, ERKs p44 and p42 MAPKs and  **CaMKII**  and the phosphorylation of c AMP responsive element binding protein  **CREB**  in the hippocampus.  Ref: 11040265 Learn MemLearn Mem, 1998
- The results indicate that CaMKIV is much more potent than  **CaMKII**  in activating  **CREB**  in three different cell lines.  Ref: 7958915 Genes Dev, 1994
- These results suggest that calcium calmodulin dependent protein kinase II  [ **CAMKII** ]  induced phosphorylation of  **CREB**  may be involved in regulating feeding behavior induced by NPY.  Ref: 9106624 Mol Pharmacol, 1997
